# What Do We Know about Pruritus in Very Young Infants? A Literature Review

**DOI:** 10.3390/cells10102788

**Published:** 2021-10-18

**Authors:** Camille Le Pors, Matthieu Talagas, Claire Abasq-Thomas, Séverine Henry, Laurent Misery, Jean-Michel Roué

**Affiliations:** 1 Department of Neonatal Medicine, Brest University Hospital, F-29200 Brest, France; jean-michel.roue@chu-brest.fr; 2 LIEN—Laboratoire Interactions Epithélium Neurones—EA 4685, Brest University, F-29200 Brest, France; matthieu.talagas@chu-brest.fr (M.T.); claire.abasq@chu-brest.fr (C.A.-T.); laurent.misery@chu-brest.fr (L.M.); 3 Department of Dermatology, Brest University Hospital, F-29200 Brest, France; 4 EthoS (Éthologie Animale et Humaine) Laboratory—UMR 6552, Rennes University, F-35000 Rennes, France; severine.henry@univ-rennes1.fr

**Keywords:** pruritus, infants, itch, atopic dermatitis, skin development

## Abstract

In infants, pruritus is frequently considered as absent because they do not scratch themselves. Because pruritus could induce severe adverse effects in this vulnerable population, we aimed to review existing evidence on the ability of young infants to experience itch and on how to assess itch-related discomfort in this population. A literature review was performed (Pubmed, Google Scholar). Neurological itch pathways are well described. Skin development starts early during gestation. At 34 weeks of gestation, skin is almost complete while skin adaptations occur after birth. Newborn skin is neurologically functional, including the ability for young infants to feel pain. Similarities and interactions between pain and pruritus support the hypothesis that infants could feel pruritus. However, the existence of pruritus in infants has never been evidenced. Many itchy conditions can affect them, suggesting non-negligible prevalence of infant pruritus among which atopic dermatitis (AD) is the most studied disease. Studies reported a negative impact of AD on children and their families. There is no existing validated method to assess pruritus in infants, although they may feel pruritus and chronic pruritus can lead to serious adverse effects. To appropriately diagnose pruritus appears of great interest among young infants. Development of a method is required to this aim.

## 1. Introduction

Pruritus (itch) is an unpleasant sensation that leads to the need to scratch. This definition has been established since the 17th century but research became dynamic in the 1990s. Nowadays, the neurophysiology of itching is well described but many concepts remain to be explored, particularly among children. In contrast, pain has been deeply studied in children, especially in infants. Although recently, it is now established that infants are competent in pain experience. In contrast, pruritus experience in infants is frequently denied because infants do not scratch themselves. Indeed, scratching movements are not observed in infants, especially before 6 months of age, while manual approaching and reaching behaviors, which are a prerequisite for scratching, are not effective before 5–6 months of age. In this vulnerable population, discomfort related to pruritus could induce serious adverse effects, particularly on neurodevelopment. Thus, characterizing and assessing pruritus in infants is of great interest.

Our aim was to review existing evidence on the ability of young infants (i.e., before 6 months old) to experience itching and on how to assess itch-related discomfort in this population.

## 2. Materials and Methods

A literature search using Pubmed and Google Scholar was performed using following key words: “pruritus”, “itch”, “infant”, “newborn”, “neonate”, “physiology”, “pain”, “assessment”, “quality of life”, “impact”, “sleep”, “atopic dermatitis”. Search was limited to English-languages articles published between January 1980 and July 2020. Titles and abstracts were screened for relevance. When they were equivocal, full text was read. Reference lists of identified articles were searched for additional articles of interest. A total of 33 articles were retained for this narrative review. 

## 3. Results

### 3.1. Neurophysiology of Pruritus—Link between Pain and Pruritus

**Figure 1:**
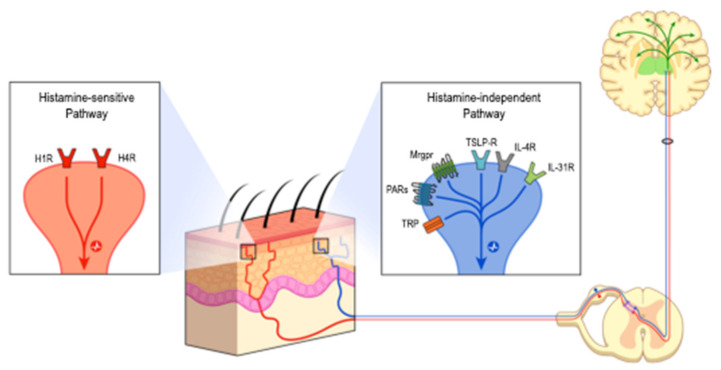
Itch processing. Itch perception is initiated in the skin by intra-epidermal nerve endings, which are the dendritic extremities of primary sensory neurons located in trigeminal and dorsal root ganglia. These intra-epidermal nerve endings mainly belong to C-fibers. The binding of chemicals’ membrane receptors leads to the activation of two major pathways: either the histamine-sensitive (histamine receptors 1 and 4) or histamine-independent (PARs, Mrgpr, TSLP receptors, IL 31 receptor, TRP).

The neurophysiology of itching is more and more well described [1,2,3,4] (Figure 1). Pruritogens activate receptors on primary sensory neurons. Nerve endings involved in pruritus are mainly C-fibers located in the epidermis. C-fibers can be separated into two itch pathways: a histamine-sensitive pathway, which involves histamine-receptors 1 and 4 (H1R, H4R), and a histamine-independent pathway with G protein-coupled receptors (GPCRs), cytokine receptors, transient receptor potential (TRP) ion channels and voltage-gated sodium channels. GPCRs include, among others, protease-activated receptors (PARs) and Mas-related G protein-coupled receptors (Mrgprs). Cytokine receptors are more particularly IL-31 receptor (IL-31R), thymic stromal lymphopoïetin (TLSP) receptor, IL-13 receptor (IL-13R) and IL-4 receptor (IL-4R). 

Primary sensory neurons terminate in the spinal cord dorsal horn, where itch information is regulated by excitatory or inhibitory interneurons, before being transmitted to projection neurons. Neurotransmitters are substance P, glutamate, natriuretic peptide B, gastrin-released peptide, brain-natriuretic peptide, neuromedin B and calcitonin gene-related peptide, for example. Their distinctive features contribute to polymorphism and redundancy in the itch circuit. 

Projection neurons relay itch signals to the thalamus or parabrachial nucleus. The signal is finally projected to different brain areas such as the primary and secondary somatosensory cortex, cingulum, insula, premotor area, supplementary motor areas, striatum, prefrontal area, cerebellum and precuneus, providing plurimodality of the itch. Histaminergic and non-histaminergic itch signals share some brain areas but also activate specific areas. 

The inhibition and excitation processes are described, involving interneurons of dorsal horn, glial cells and midbrain periaqueductal gray. 

Researchers report a sensitization phenomenon in pruritus, leading to allokinesis and hyperkinesis. Sensitization occurs at peripheral level, involving nerve growth factor (NGF), artemin, prostaglandins and PAR-2, and at central level in spinal cord and brain. Those sensitization patterns are strikingly similar to those of pain. Allokinesis and hyperkinesis are analogous of allodynia and hyperalgesia in pain. Actually, itching and pain share many similarities [1,2,5,6]. They use similar mechanisms to transduce their signal from peripherical nerves to integration centers and have many common receptors and mediators (PAR, TRP, NGF, substance P, prostaglandins, serotonin, opioids, cannabinoids). The involved brain areas are not strictly separated: there is a large overlap in involved integration centers. 

As in chronic pain, hypersensitivity to C-fibers input exists in chronic itching: a normally painful stimulation is perceived as an itch in patients with atopic dermatitis (AD). Thus, sensitization can lead to the loss of pain-induced inhibition in patients with chronic itching. 

Itching and pain interact with each other. It is well known and experimentally proved that scratching-induced pain inhibits and relieves itching [1]. Inversely, analgesia can enhance an itch: µ-opioid agonists administered in spinal cord induced segmental analgesia and itching [1]. Some researchers also described an itch induced by nociceptors activation, but further investigations are needed [2]. 

Therefore, regarded as the “little brother of pain” for a long time, pruritus benefited from growing literature during the last few decades [2]. Pain and itching are distinct sensations, with different pathways and reflex patterns, but they are not strictly independent, sharing similarities and interactions. Concurrently with pruritus research, research on skin development in infancy has been prolific.

### 3.2. Newborn Skin: From Intra-Uterine Development to Neonatal Adaptation

**Figure 2:**
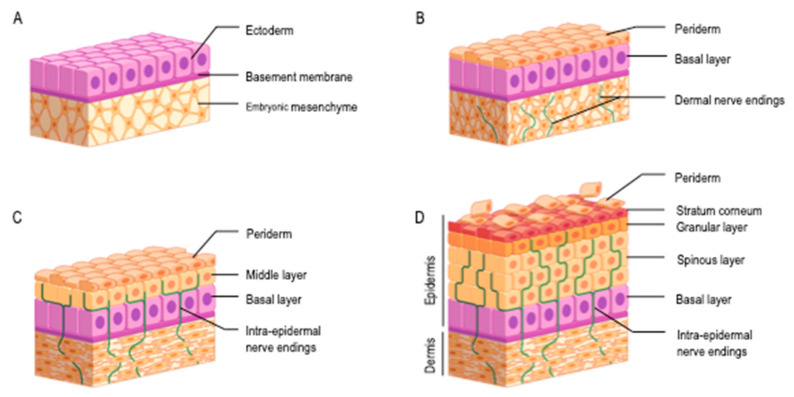
Skin development. (**A**) End of the 1st month of gestation. The epidermis and the dermis derive from the ectoderm and the embryonic mesenchyme, respectively; (**B**) two months of gestation. The periderm covers a basal layer and emergence of dermal nerve endings; (**C**) end of 3rd month of gestation. The periderm covers middle and basal layers; emergence of intra-epidermal nerve endings; (**D**) from the end of 4th month of gestation. The epidermis is made of basal, spinous and granular layers covered with the stratum corneum. The periderm peels off during the second part of intra-uterine development.

The epidermis derives from the ectoderm and its development begins early during gestation [7,8,9,10,11] (Figure 2). Cells differentiate according to differentiation programs involving Wnt, fibroblast growth factor (FGF), bone morphogenetic proteins (BMP) and Notch pathways [12].

Primarily, a single layer of ectoblastic cells covers an embryo’s body. At 4 weeks of gestation (WG), the epidermis is made of two layers: basal layer and periderm [12,13,14]. Periderm, covering the basal layer, is a transitory structure which protects the fetus against amniotic fluid. Between weeks 10 and 12, basal layer proliferates in an intermediate layer. Epidermis cells proliferation results in epidermis stratification: between weeks 14 and 17, there are 6–8 basal layers for each periderm layer and upper cells are flattened. From WG 23, periderm is absent, the outermost layer is the stratum corneum (SC). Epidermis is fully keratinized at 26 WG and composed of a basal layer, 2–3 spinous layers, a granular layer and 5–6 SC layers (from depth to surface).

Specific cells appear sequentially. Melanocytes, developing from the neural tube, appear in the basal layer during the fifth week, Langerhans cells from the sixth week. At 8 weeks, keratinocytes emerge from the basal layer to form a third layer between previous ones: the spinous cells layer. Between the second and the third month of development, Merkel cells appear. They are of epidermal origin. Mast cells are described around the 11th week. 

SC is often described as a wall made of “bricks” (corneocytes) and “mortar” (lipids i.e., sterols, free fatty acids (FFA), ceramides, phospholipids) [15]. This architecture confers the SC its vital barrier functions to water loss and substance penetrance [16]. Proteins are essential for epidermis integrity, especially keratins [12]. Other proteins include filaggrin: it aggregates keratins [17] and is proteolyzed to form natural moisturizing factors (NMF), which play a major role in skin hydration and permeability [12].

Below the epidermis, separated by the basement membrane zone, dermis derives from the mesoderm [14]. At 6 weeks, it consists of loose connective tissue with small non-branching blood vessels.

At later ages, dermis becomes denser in connective tissue and the number and branching of blood vessels increase [9]. Dermis differentiates during the third and fourth months into a connective tissue containing elastic and collagen fibers, the chorion. It forms numerous irregular papillary expansions, the dermal papillae, in contact with epidermis. At WG 13, dermis consists of fine fibers and many cells. The expression of dermis structural proteins, i.e., collagen, fibronectin, chondroitin sulfate and elastin, differs from fetal to adult skin [18]: higher levels of collagen III, chondroitin sulfate, proteoglycans and hyaluronan as compared with adult dermis; contrarily, elastin is absent in fetal dermis. In later gestation, dermis thickens by an increase in collagen content. Hair pegs appear around WG 16 [18]. 

From WG 6–7, the fetus is able to respond to external stimuli (light touch, blood sampling) [9,19,20]. Sensory nerve endings grow during fetal development. At WG 6, nerve fibers are seen in dermis as sub-epidermal plexuses. Between WG 10 and 12, the number of dermal fibers increases and intra-epidermal nerve fibers appear from sub-epidermal plexuses [9]. Sensory cutaneous receptors are described in the dermis and epidermis early in gestation [21,22]: Merkel cells and Pacinian corpuscles appear during the first trimester, Meissner corpuscles in the early third trimester. Contacts between Merkel cells and nerve endings exist from 8–9 WG to form Merkel cell–neurite complexes [22], Hand innervation has been specifically studied [21,23]: most of hand surface is covered by nerves at WG 8.5 and topography appears mature by WG 11.5. During the late embryonic–early fetal period, i.e., around 8–10 WG, an adult-like pattern of the skin domain covered by each nerve is established. In later development, remodeling of sensory innervation occurs. 

At 34 weeks, skin is structurally almost complete. Its neurological apparatus is effective but still maturing. After birth, skin continues evolving notably in its physiological functions. Adaptations and maturation processes are described: variation in transepidermal water loss (TEWL) which reflect the setting-up of skin barrier function [12,24,25,26,27], changes in skin hydration and water-holding capacities [12,26,27,28], skin pH variation [24,27,28], sweat and sebaceous glands functioning [15,24,29], development of the skin innate immunity role through proteins, lipids and immunity cells and microbiome [12,13]. 

### 3.3. Neurosensory System and Infant Skin Features Bring Clues in Favor of Infants’ Ability to Experience Pruritus

Therefore, newborn skin is immature and constantly evolving but it is functional, notably in its neurophysiological development, with strong evidence that newborns experience pain during skin-breaking stimuli [6,30].

Cutaneous sensory receptors appear early in gestation and are present on all cutaneous surfaces by the 20th WG [19,30]. At a central level, the architecture of the dorsal horn of the spinal cord develops before 13 WG and is complete at 30 WG: interneurons develop, synaptic connections and neurotransmitters vesicles become organized [30]. Cerebral cortex grows between the 8th and the 24th WG with neuron development, dendritic processes and synaptic connections [30,31]. 

Neurotransmitters associated with pain perception, such as substance P and neuromedins, are identified in the central nervous system from 12 WG. The endogenous opioid system is functional at birth and high levels of beta-endorphins are measured in response to stressful situations. 

In a functional perspective, neonate neurosensory maturity is proved by changes in electroencephalogram during tactile stimuli, by metabolic activity (e.g., glucose consumption) in brain sensory areas (i.e., sensorimotor area, thalamus) and by identification of cognitive, coordinative and associative skills [19,30,31].

Those anatomic, chemical and functional bases of pain perception in newborns are common with that of pruritus, as described previously. Thus, it seems likely that newborns and older infants are competent for itch experience. This hypothesis is reinforced by analogous mechanisms and functional interactions between pain and itch systems. In addition, beta-endorphins variations are described in response to itch and beta-endorphin levels reflecting itch intensity [32].

There are very few studies and evidence on the experience of pruritus in young infants. Motor skills development [33] and verbal abilities do not allow infants under 6 months of age to express usual behaviors in itchy conditions. Pruritus in this population is not recognized and is even frequently denied by doctors although itchy conditions can affect young infants.

### 3.4. Pruritus during Infancy: Etiology, Epidemiology

In adults [34,35,36,37,38], the prevalence of itching is 8–22% in the general population depending on studies, with a trend in increasing prevalence. There is no epidemiological study about child pruritus, although many diseases can cause itching in children including dermatological and systemic diseases [2,39,40]. The most frequent causes of pruritus are dermatoses, especially AD. Characterized by intense and chronic pruritus, AD is diagnosed during the first six months of life in 45% cases, usually around the age of 3 months [40]. Other itchy dermatoses are urticaria, cutaneous infections, zoonoses such as scabies for the most frequent, and to a lesser extent, psoriasis, mastocytosis, autoimmune diseases (bullous pemphigoid, herpetiform dermatitis) and photodermatoses, among others.

Systemic causes of pruritus are rarer [2]. They include hepatic diseases (biliary atresia, cholestasis), terminal renal failure, hematologic and neoplastic diseases (Hodgkin’s disease, tumors of the central nervous system). Drug-induced pruritus should be very uncommon in infants.

Beside skin and systemic diseases, genodermatoses can cause pruritus: ichthyosis (Sjogrën-Larsson syndrome), Netherton syndrome, epidermolysis bullosa, Costello and cardiofaciocutaneous syndromes and neurofibromatosis type I.

Even if there are no data on pruritus prevalence in infants, etiologies are various. This suggests a non-negligible prevalence of infant pruritus.

### 3.5. Impact of Pruritus: The Example of Atopic Dermatitis (AD)

There is no specific literature about infant pruritus and its impact. Conversely, the impact of AD, the most common chronic disease in children with pruritus as a major symptom, has been deeply studied, affecting up to 20% of children [41,42,43]. AD is an inflammatory skin disease characterized by skin and systemic inflammation and barrier dysfunction. The clinical presentation varies depending on age, ethniticy and the underlying biological mechanism [44]. However, pruritus is the first cause of morbidity in AD [42,43,45]. No study was conducted in young infants, while they could also deeply suffer from itching and its consequences.

Children with AD rate itching and sleep disturbances as the most disabling symptoms [46]. Sleep disturbances include difficulties falling asleep, night awakenings, reduced sleep duration and efficiency, awakening difficulties and daytime sleepiness [46,47]. Parents attribute them to pruritus [47]. Objective measurements confirm self- and parent-reported impacts: children with AD lose an average of 2 h sleep per night [41]. Sleep abnormalities are frequent during infancy but are more prevalent in children with AD, especially during flares, and can persist during remissions, resulting in chronic sleep disorders [46,47]. Sleep difficulties in AD are associated with behavior and emotional troubles: irritability, mood changes, fussiness, tiredness, clinginess, crying, attributed by parents to pruritus [46,47]. Sleep disturbances correlate with mental health problems: AD during infancy is associated with increased prevalence of attention deficit hyperactivity disorder (ADHD) [43,45]. Children with AD are also more at risk of depression, anxiety, conduct disorder and autism. Underlying mechanisms are unclear, but it is widely accepted that sleep loss due to pruritus can affect brain development and promote mental health disorders. Other mechanisms could include effects of cytokines on the neuroimmune system, effects of psychological stress and effects of the chronic nature of the disease. Such mechanisms are not specific of AD and exist in other pruritic affections.

Social functioning and school performances are affected by pruritus [41,42,46,47]. Although infants are not concerned, this highlights potential adverse effects of pruritus in this younger and vulnerable population.

Children’s quality of life (QoL) is deeply impaired. The more severe AD is, the greater QoL decreases [42]. AD causes the greatest burden on QoL among all chronic diseases (diabetes, asthma, enuresis, cystic fibrosis) except for cerebral palsy. Among skin diseases, only scabies and psoriasis, which are very pruritic conditions, have a greater QoL impact [42,46].

Families’ QoL also decreased with a child with AD [42]. Impact on family correlated with AD severity [41]. Sleep deprivation is the most reported impact: 60% for parents, 38% for siblings. Parents can lose up to 2.5 h sleep per night, notably because of co-sleeping, a strategy to prevent child scratching and awakening, used by 30% of parents [41,42,46,47]. It results in parental exhaustion added to reported feelings of guilt, emotional distress, helplessness, worries about child development, spousal altered relationship, social isolation and anxiety about strangers’ reactions [41,46,47]. The financial burden of AD affects family QoL [41,46]. Direct (medical visits, treatment…) and indirect (time away from work, lifestyle changes…) costs are difficult to estimate (specifically in young infants due to the lack of precise measures) and vary according to disease severity and health care systems, but the estimated cost of AD is considerable: up to USD 821 of direct cost annually per patient in the US and up to USD 4.228 billion per year for the US economic system.

Therefore, AD’s impact is considerable in children and their families. Although studies were mostly conducted in children after one year of age, this suggests a high impact in younger infants.

### 3.6. Assessment of Pruritus in Young Infants: A Need for Further Research

Young infants’ skin and sensory nervous systems appear functional for pruritus experience. Various itchy diseases can affect infants, but pruritus is subjective, and thus difficult to diagnose and assess [48], particularly in this non-verbal population. Many tools were developed to assess pruritus and its associated burden [49,50]. A Visual Analogue Scale, Numeric Rating Scale, Verbal Rating Scale, 5-D Itch Scale and Itch-Severity Scale are the most used. Nevertheless, they are not validated in children under 7 years. They are mostly based on self-reported items not transposable to a non-verbal population. Parent-reported items raise the risk of measurement bias. Moreover, such methods have the limitation that itching and scratching may be unconscious. Reliable measurements are needed to better assess itching in infants.

Itching, as a subjective sensation leading to the need to scratch, could be indirectly assessed based on scratching behaviors. Studies were conducted to objectively measure scratching during sleep [51]. Researchers evaluated movement transducers attached to bed legs, measurement of forearm muscle potentials, wrist pressure-sensitive meters, electromagnetic movement detection, paper strain gauges applied to the wrist, piezoelectric devices applied to fingernails and direct videotaping of subjects. However, studies were almost always conducted in the adult population and results were inconclusive. Moreover, such methods are time-consuming and available only in a hospital setting. Therefore, these instruments are not used in daily practice and infants do not scratch themselves.

Some authors use actigraphy (wrist accelerometers) during the night to measure scratching in children [51]. Actigraphy is more convenient and allows home-measurement. It was validated in scratching and restlessness evaluation against the gold standard of infrared videotaping and seems to reflect scratching severity and sleep disturbances. However, the population studied was over 2 years old, and samples were small and mostly diagnosed with AD, thus generalizability is weak. In addition, sleep patterns in neonates are not correctly assessed by actigraphy, questioning the reliability of this tool in the population of young infants [52]. Moreover, sleeping hours are long in this population; this sleep profile could affect their itching experience. Therefore, actigraphy must be specifically assessed in young infants to determine its reliability in itch evaluation.

Specific challenges related to young infants exist. First, motor skills development does not allow infants less than 6 months old to appropriately scratch themselves. On one hand, this may break the itch–scratch vicious circle and reduce pruritus. On the other hand, this may lead to an underestimation of pruritus-related discomfort by considering itch does not exist (alike pain was denied among neonates in the past). Secondly, all itching sensations do not lead to scratching movement (self-impeachment, avoidance strategies). Finally, sleep disturbances are common in infants and may not reflect pruritus. Similarly, every limb movement does not reflect scratching and an itching sensation can be expressed through rubbing or writhing instead of scratching. Therefore, actigraphy reliability to assess pruritus in infants is questioned. Another limitation of these studies is the measurement variability in the same subject from night to night. It was attributed to variation in symptom severity, but authors could not exclude measurement error. Moreover, environmental confounding factors (humidity, temperature) were not considered. Lastly, although wrist accelerometers are more convenient than previously cited instruments, feasibility and acceptability remain poor in clinical practice.

In AD, some authors tried to identify serum and cutaneous biomarkers of itching [32]. Serum chemokines levels correlated with AD severity but not with pruritus, whereas serum IgE, ß-endorphins and TEWL correlated with itch intensity. However, these studies were not specific to infants, and therefore results might not be applicable to their evolving skin. Moreover, such assessments require invasive procedures that could be avoidable.

## 4. Conclusions

To diagnose pruritus and appropriately assess related discomfort appears to be of great interest among young infants: many itchy conditions can affect infants and associated pruritus can lead to serious adverse effects in infants and their families.

Although young infants are probably competent in pruritus experience, there is no existing validated method to diagnose itch and its impact in this population. Self-reported scales are unusable in this non-verbal population and parent-reported tools are at high risk of subjectivity, with low reliability.

Pain assessment faces the same difficulties. Responses generated by newborns and infants during painful procedures were studied by ethologists [53,54]. Physiological (i.e., cardiorespiratory, hormonal, metabolic) and behavioral (i.e., motor responses, complex movements, facial expressions, cry) changes were identified [30,53,54,55]. Thus, appropriate pain assessment scales were developed and validated [53]. Given the closeness between pain and pruritus, studying infants’ physiological and behavioral responses in itchy situations, according to ethological methodology, could characterize itch-related signals in this population incapable of scratching because of its motor skills development stage.

## Data Availability

Data sharing is not applicable to this article.
